# Supplementary material for: How solute atoms control aqueous corrosion of Al-alloys
Source: Nat Commun. 2024 Jan 16;15:561. doi: 10.1038/s41467-024-44802-5 (PMC10792079; doi:10.1038/s41467-024-44802-5)
Supplement: Supplementary file 1 — Supplementary Information [file 41467_2024_44802_MOESM1_ESM.pdf]

Supplementary Information

How solute atoms control aqueous corrosion of Al-alloys

Huan Zhao[1,2,*], Yue Yin[1], Yuxiang Wu[1], Siyuan Zhang[1], Andrea M. Mingers[1], Dirk Ponge[1], Baptiste Gault[1,3], Michael Rohwerder[1], Dierk Raabe[1,*]

1. Max-Planck-Institut für Eisenforschung, Düsseldorf, Germany
2. State Key Laboratory for Mechanical Behavior of Materials, Xi'an Jiaotong University, Xi'an, China
3. Department of Materials, Royal School of Mines, Imperial College London, London, UK

Supplementary Figures

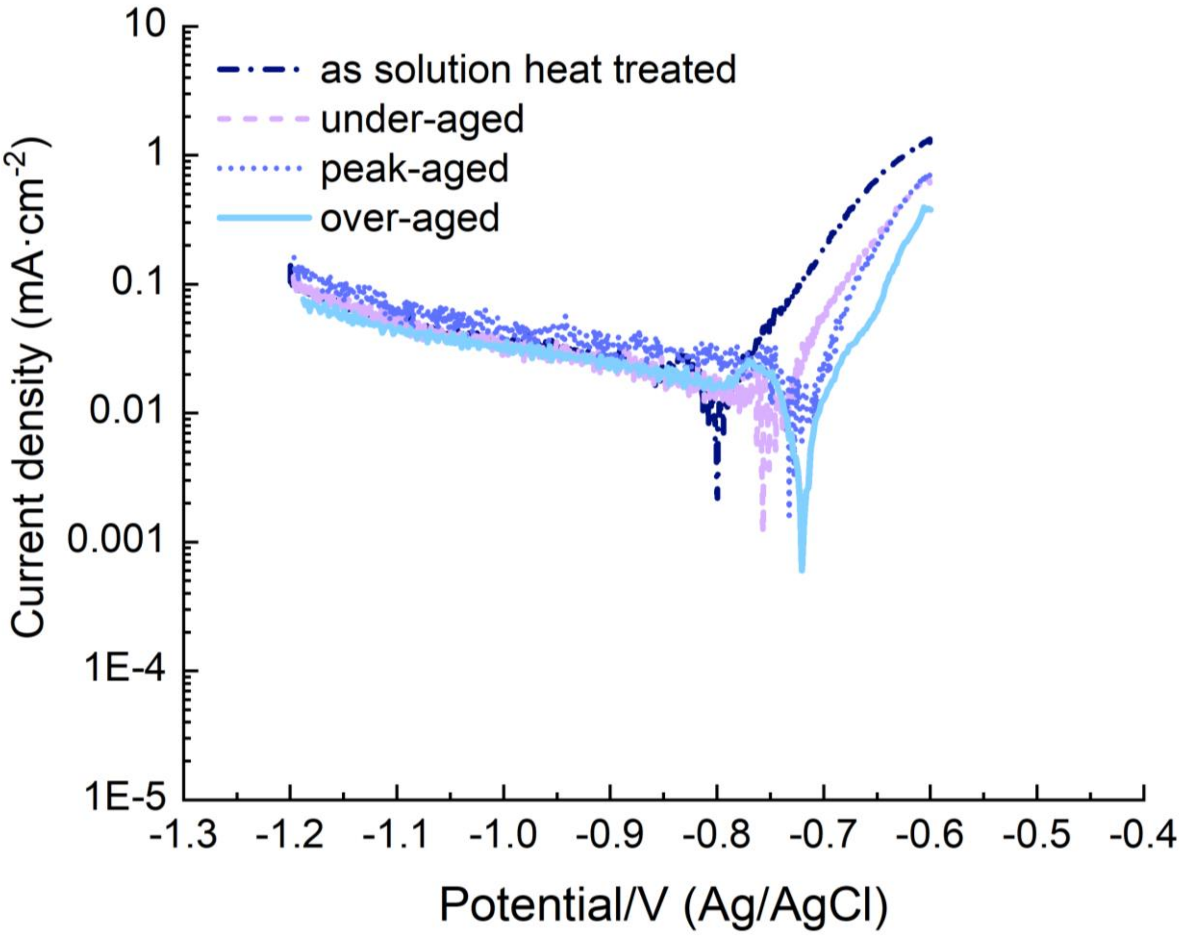

Supplementary Fig. 1. Representative polarization curves of Al-Zn-Mg-Cu alloys in deaerated 0.01 M KCl solution with a scan rate of 1 mV/s. a, As solution heat treated (475 ºC, 24 h). **b,** Under-aged (120 ºC, 2 h). **c,** Peak-aged (120 ºC, 24 h). **d,** Over-aged (120 ºC, 24 h+180 ºC, 6 h).

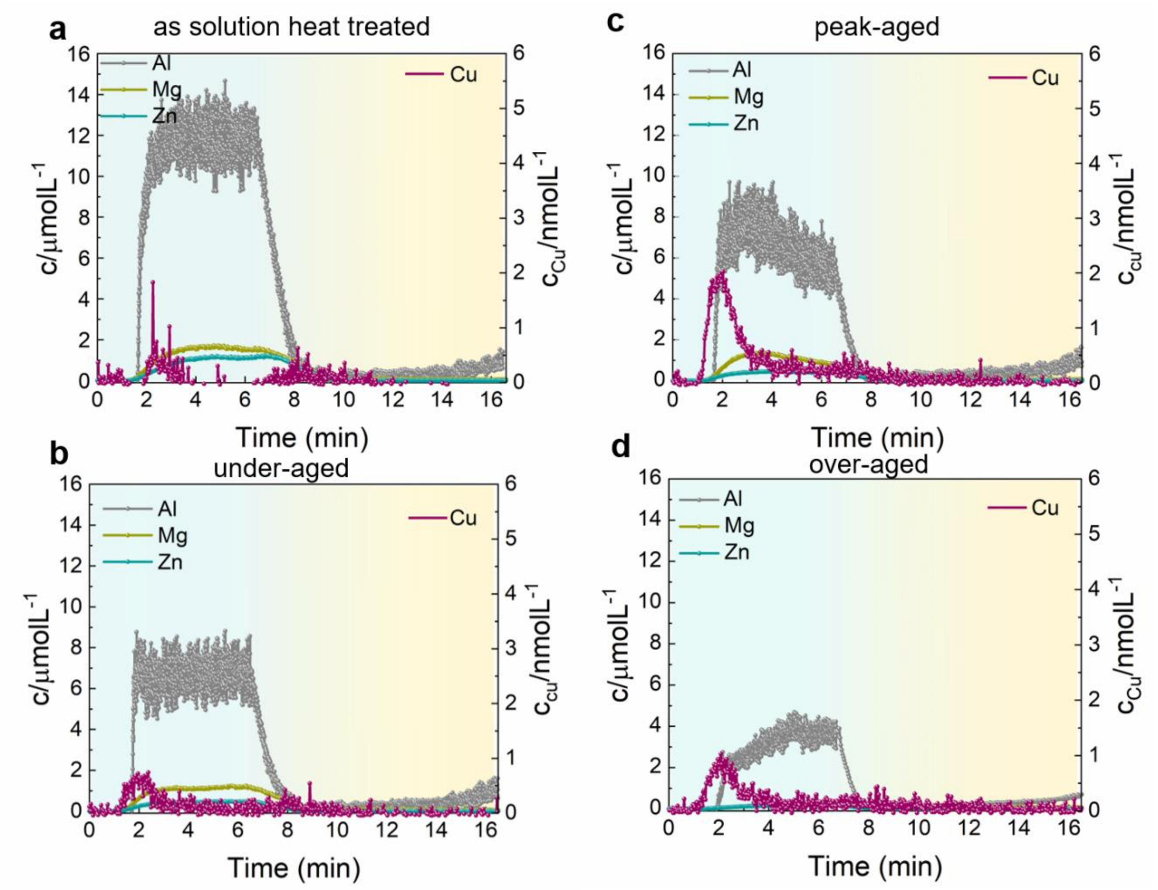

Supplementary Fig. 2. **Online ICP-MS dissolution profiles of dissolved species as a function of time. a,** As solution heat treated (475 ºC, 24 h). **b,** Under-aged (120 ºC, 2 h). **c,** Peak-aged (120 ºC, 24 h). **d,** Over-aged (120 ºC, 24 h+180 ºC, 6 h).

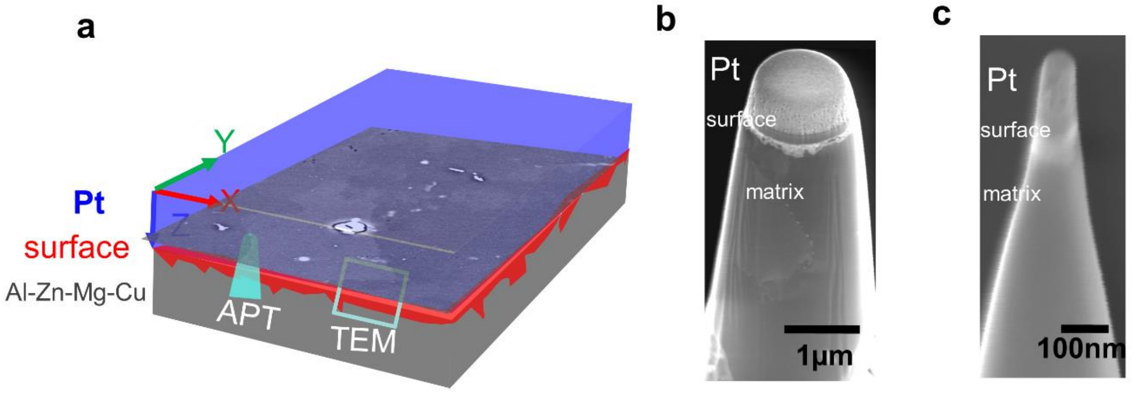

Supplementary Fig. 3. **Schematic diagram illustrating sample preparation method. a,** The side view and top-down view for APT and TEM sample preparation. **b-c**, Scanning electron images showing the oxide film formed after corrosion.

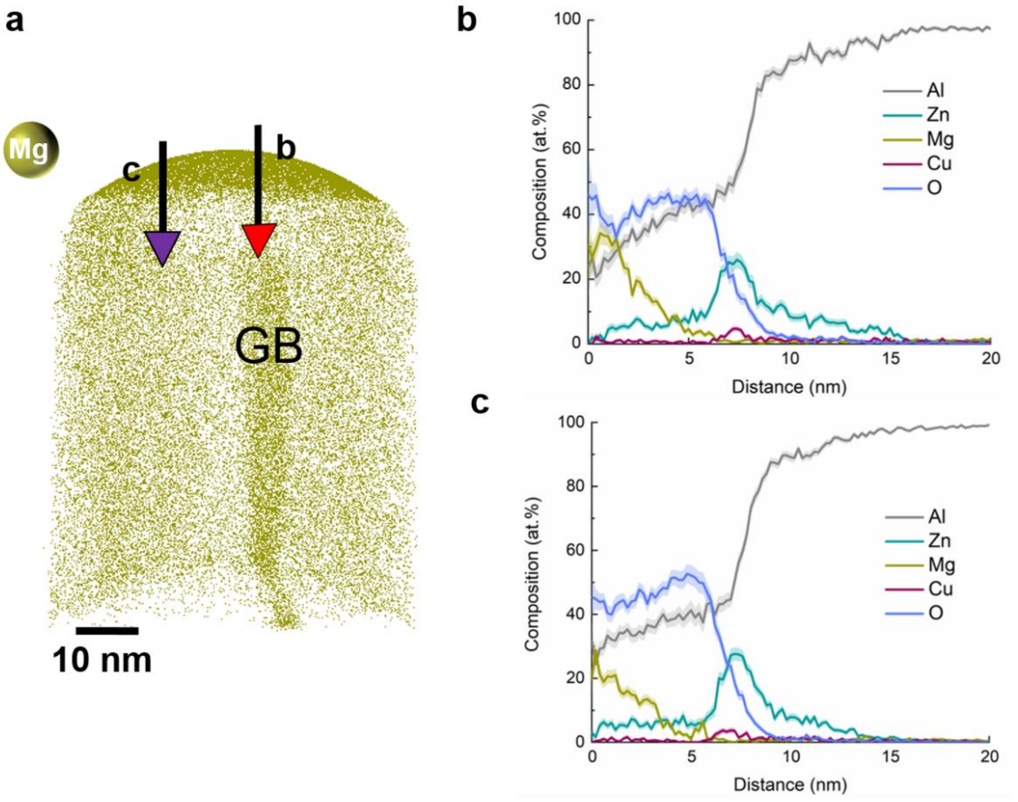

Supplementary Fig. 4. Atom probe analysis in the as-quenched Al-Zn-Mg-Cu sample after 3 hours immersion in 0.1M KCl. a, Atom map of Mg. **b-c**, The composition profile showing the composition of the oxide film: **b**, at the GB. **c**, in the bulk.

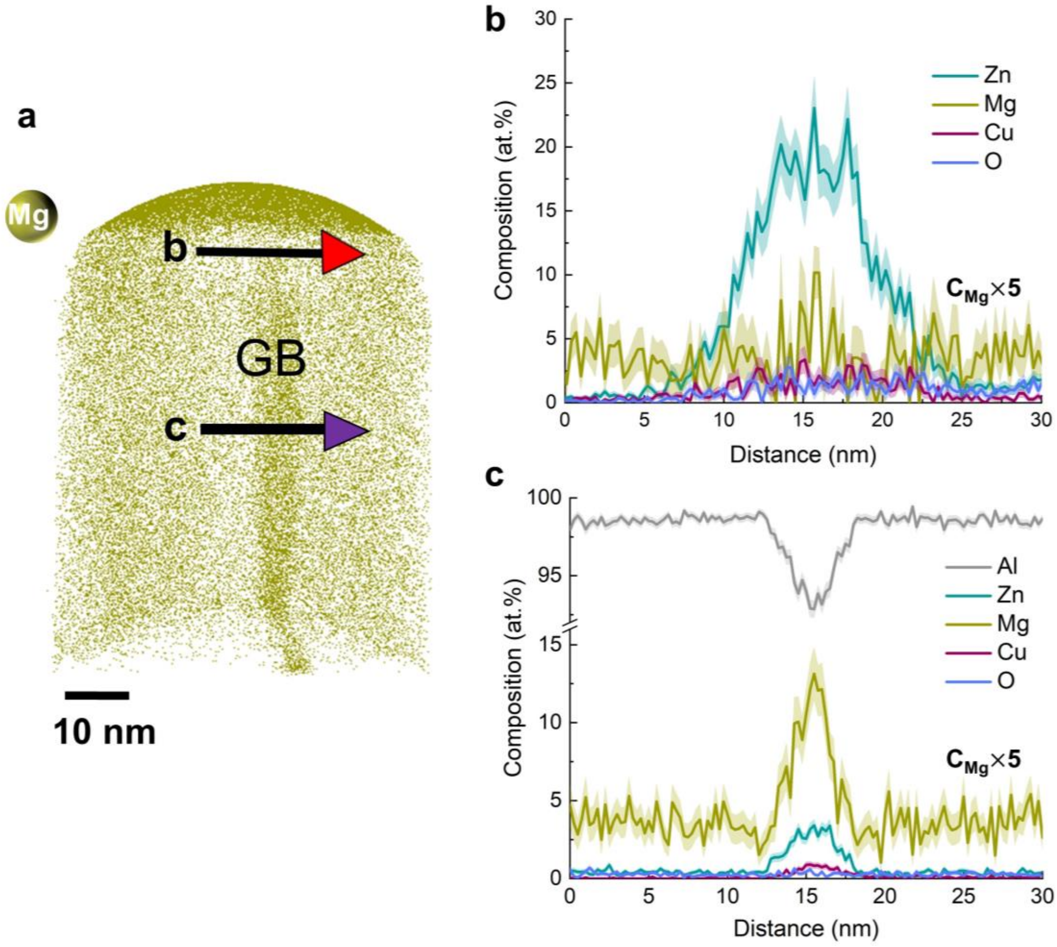

Supplementary Fig. 5. Atom probe analysis of the as-quenched Al-Zn-Mg-Cu sample after 3 hours immersion in 0.1M KCl. a, Atom map of Mg. The concentration profiles showing the GB compositions: **b**, close to the oxide. **C**. 30 nm below the oxide.

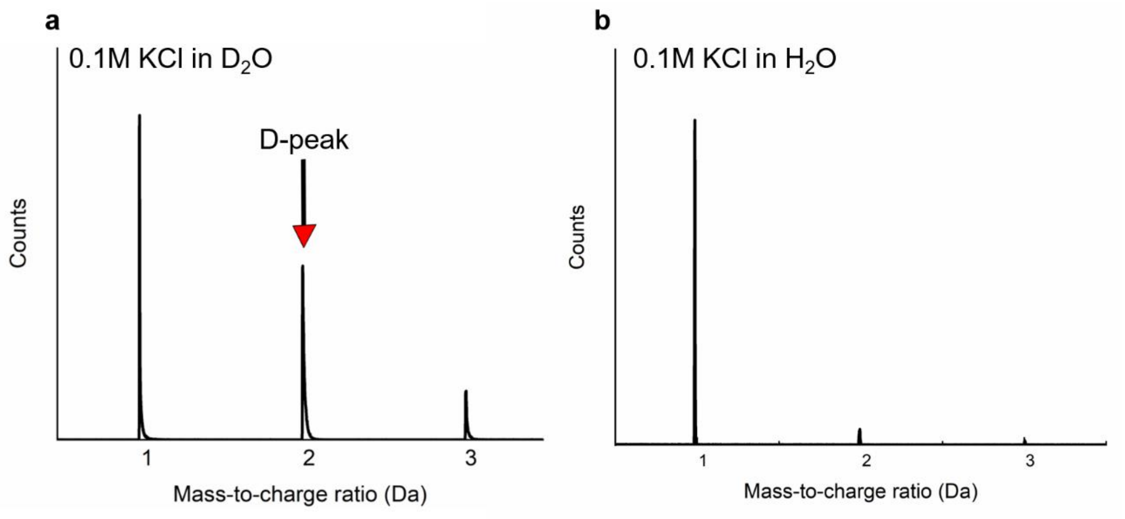

Supplementary Fig. 6. Associated H peaks in the mass-to-charge ratio of the oxide in as-solutionized Al–Zn–Mg–Cu samples. a, 0.1M KCl in D_2O. **b**, 0.1M KCl in H_2O.

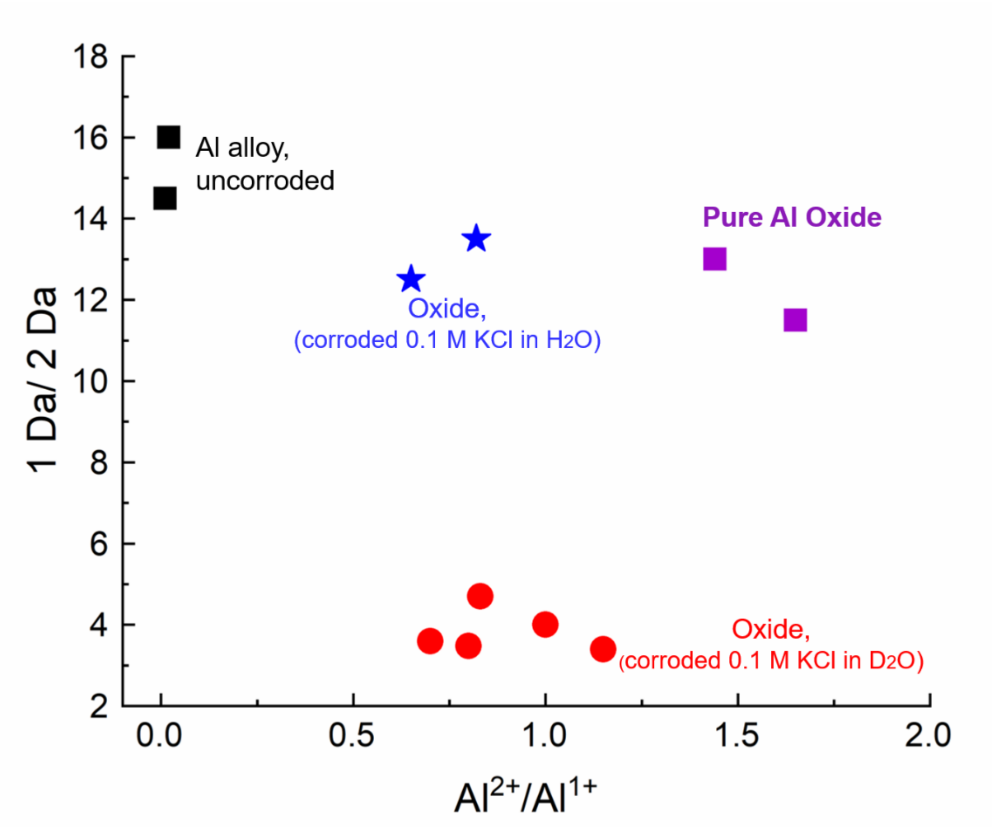

Supplementary Fig. 7. Reliability of D measurements within the oxide in APT. The ratios of 1 Da (H^+) to 2 Da (H_2^+ and/or D) within the oxide of the sample corroded in 0.1 M KCl in D_2O plotted against field strength, showing an increase with an order of magnitude of the D composition compared to reference values. Reference values include measurements on uncorroded alloy, oxide within the sample corroded in 0.1 M KCl in H_2O, and in a pure Alumina.

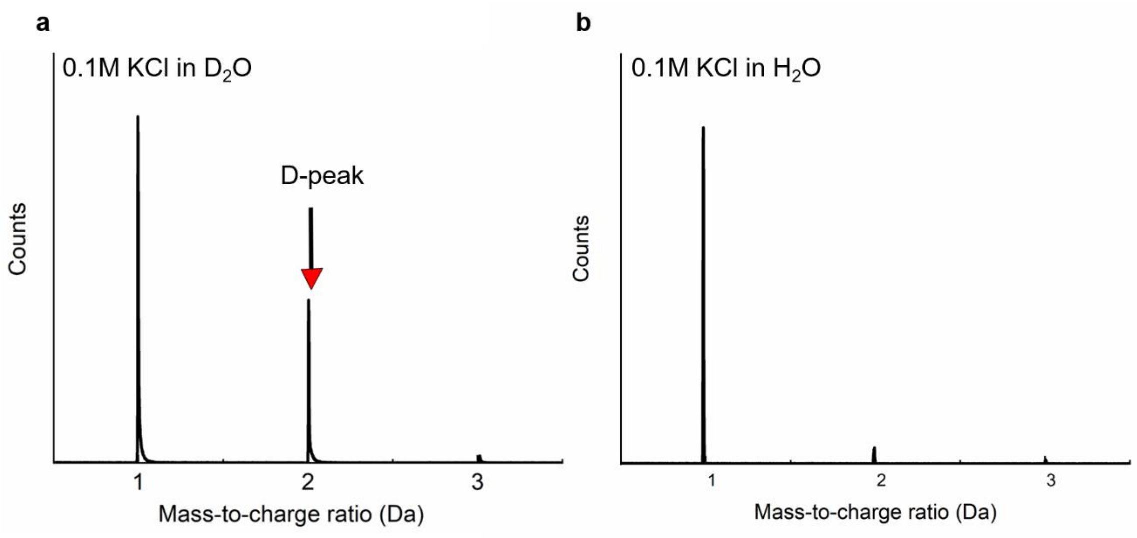

Supplementary Fig. 8. Associated H peaks in the mass-to-charge ratio for the atom probe analysis of the oxide in peak-aged Al–Zn–Mg–Cu samples. a, 0.1M KCl in D_2O. **b**, 0.1 M KCl in H_2O.

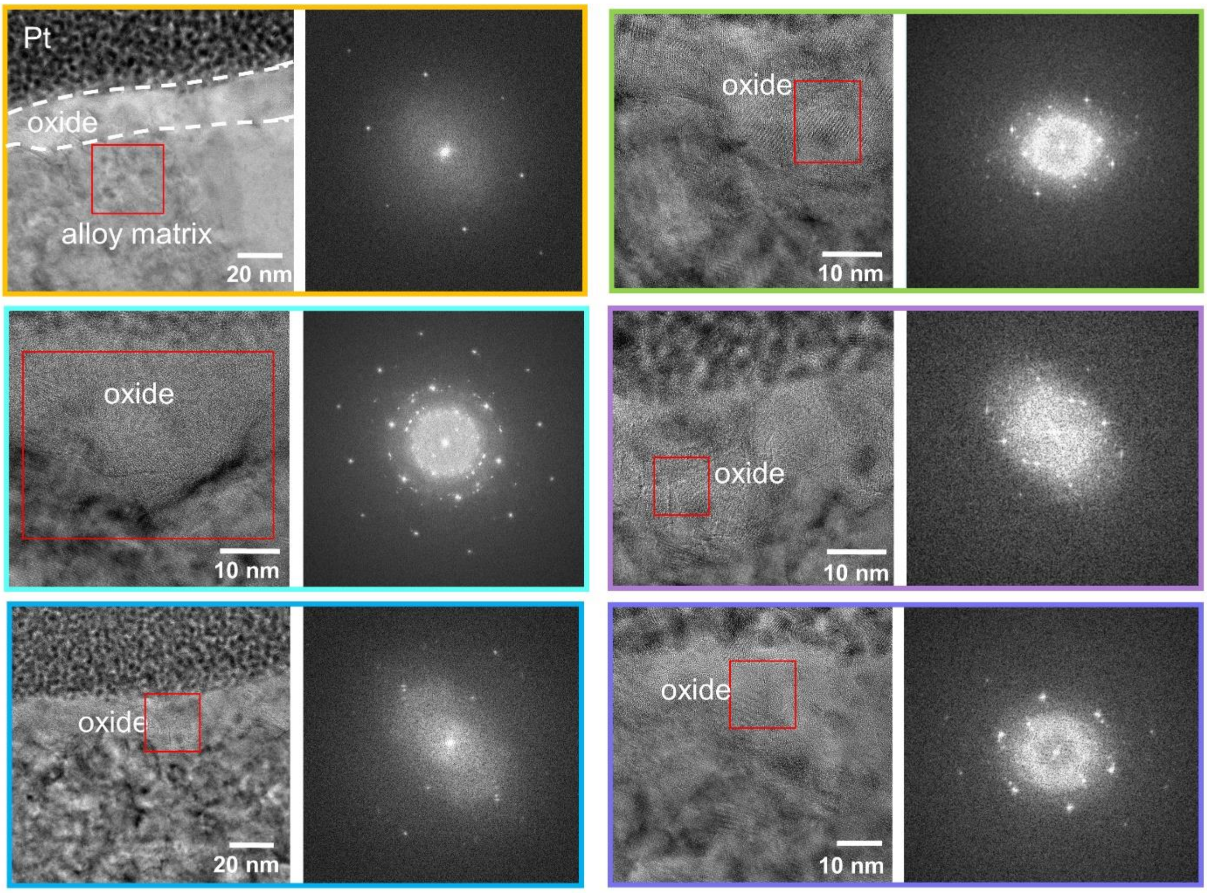

Supplementary Fig. 9. Fast Fourier transform patterns obtained from different regions of the oxide indicating the nanocrystalline structure.

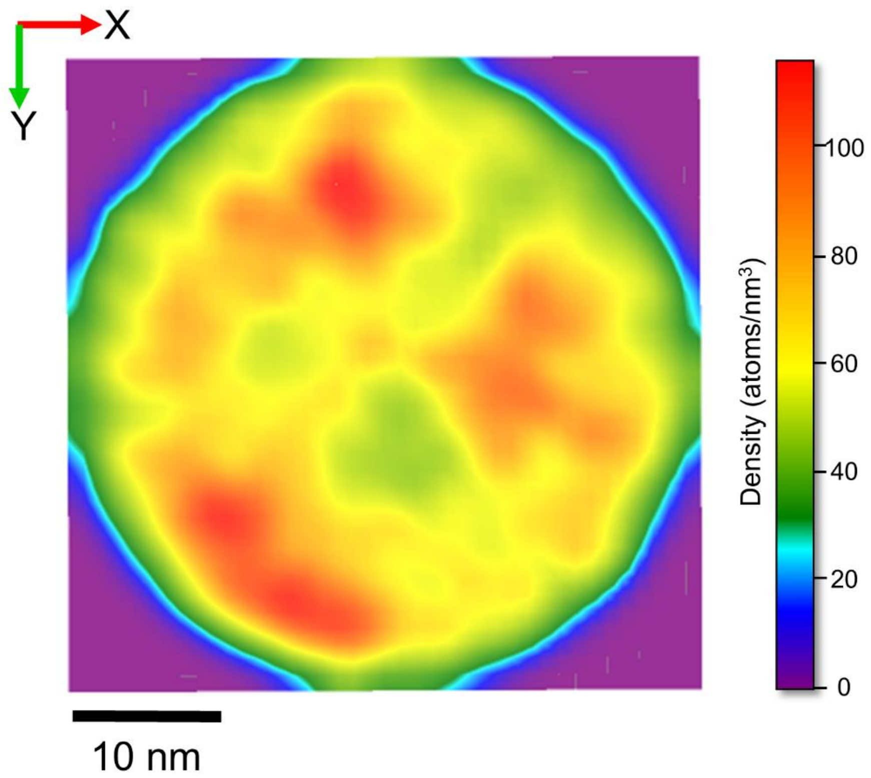

Supplementary Fig. 10. APT density map of the oxide in the peak aged sample after 3 hours immersion in 0.1 M KCl in D_2O.

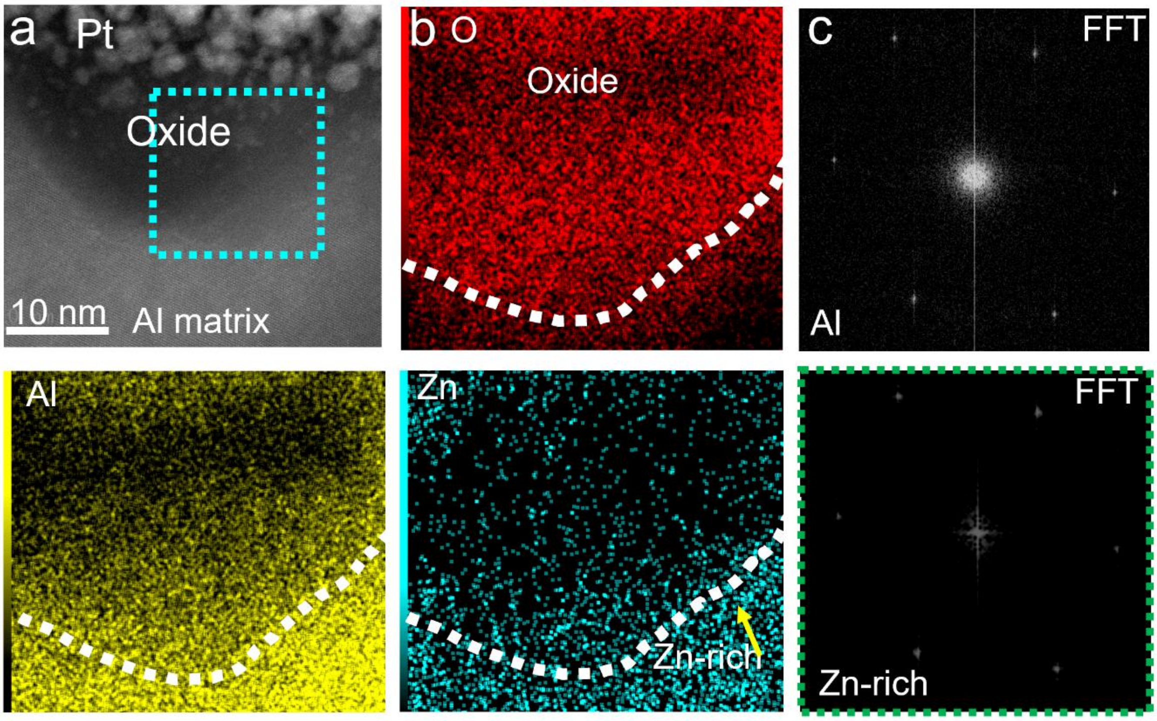

Supplementary Fig. 11. **STEM analysis of a peak-aged Al-Zn-Mg-Cu alloy after exposure to 0.1 M KCl for 3 hours. a**, Low magnification HAADF image showing the cross-section from the corroded surface (covered by Pt) to oxide and Al matrix. **b**, STEM-EDS analysis of the oxide and Zn-rich region. **c**, FFT patterns obtained from Al matrix and Zn-rich region.

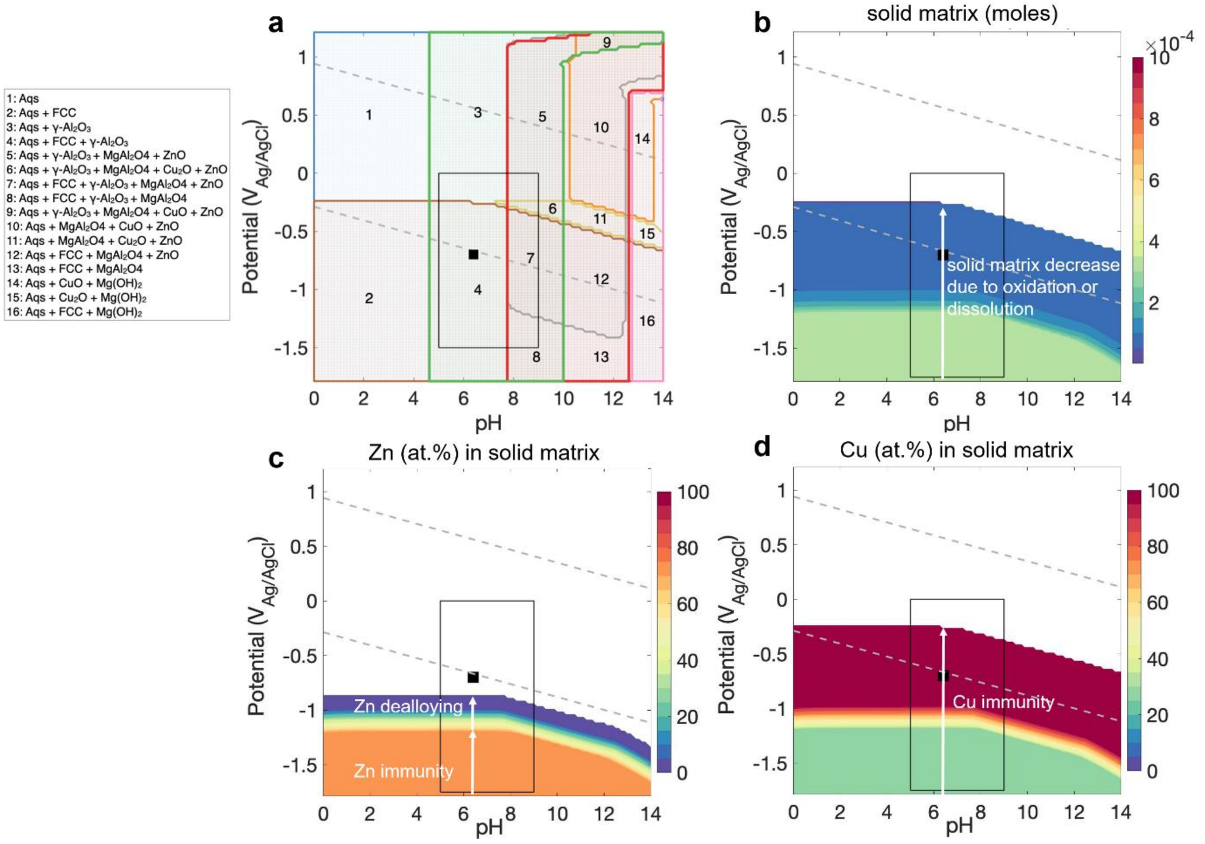

Supplementary Fig. 12. Potential-pH diagrams for the Al-Zn-Mg-Cu alloy in 0.1M KCl at 25 °C and 1 atm. a, Potential-pH diagram showing equilibrium phase regimes of oxides, aqueous solution (Aqs.), and alloy matrix (FCC). **b-d**, Potential-pH diagram showing the equilibrium dissolution characteristics of matrix phase: **b**, decrease of Al matrix content (moles) as a function of potential and pH. **c-d**, Equilibrium compositions of Zn and Cu of de-alloyed matrix as a function of potential and pH. Reversible potentials for H and O evolution with the stable region for water in between are highlighted as grey dashed lines.

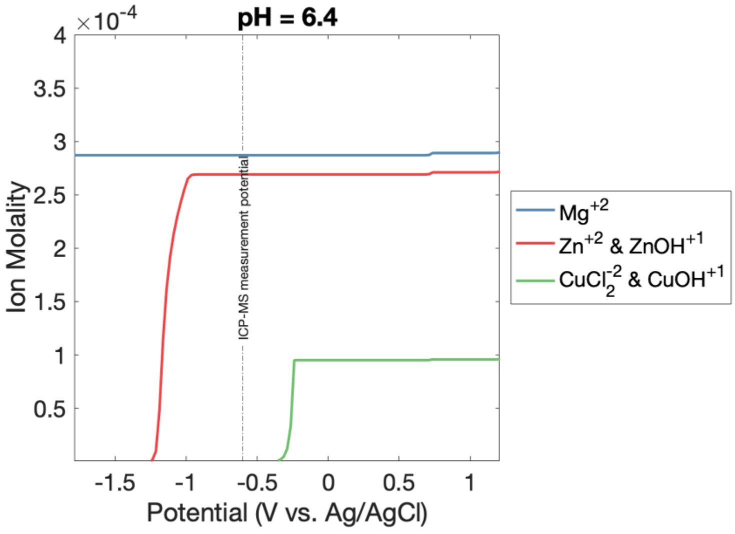

Supplementary Fig. 13. Ion concentrations (in molality) in aqueous solution at pH of 6.4. The interaction system contains 10^{-2} mol of Al–2.69Zn–2.87Mg–0.95Cu (at.%) alloy and 0.01M KCl in 1 kg water at 25°C and 1atm ambient pressure. All ions with molality larger than 10^{-6} are included in the evaluation.

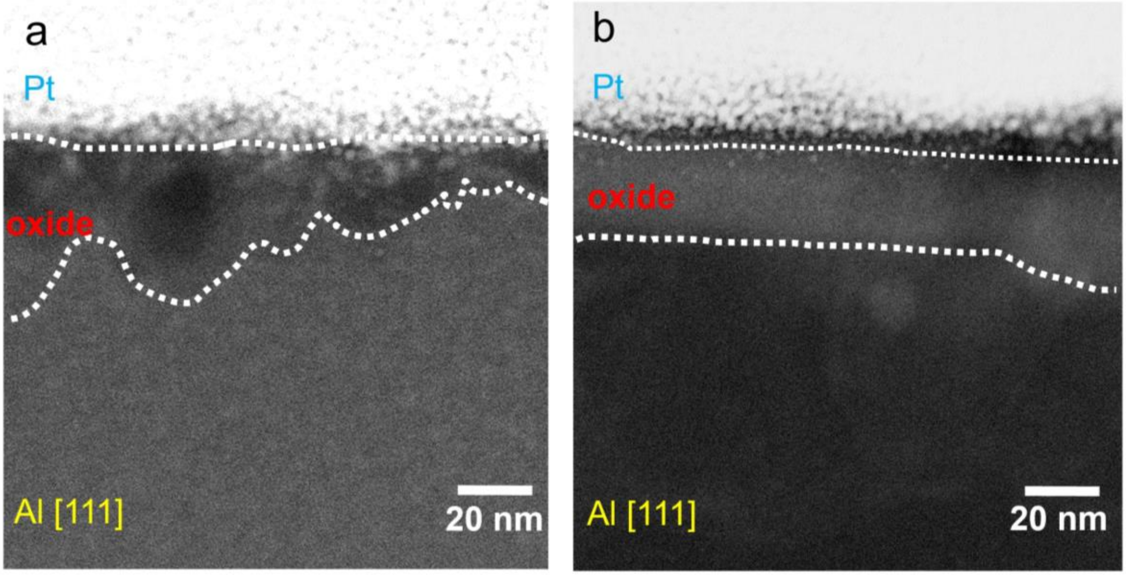

Supplementary Fig. 14. STEM analysis of Al-Zn-Mg-Cu alloy after exposure to 0.1 M KCl solution for 3 hours. a, Peak-aged **b**, Over-aged.
